# Mastering the brain in critical conditions: an update

**DOI:** 10.1186/s40635-023-00587-3

**Published:** 2024-01-05

**Authors:** Chiara Robba, Elisa R. Zanier, Carmen Lopez Soto, Soojin Park, Romain Sonneville, Raimund Helbolk, Aarti Sarwal, Virginia F. J. Newcombe, Mathieu van der Jagt, Jan Gunst, Tobias Gauss, Samy Figueiredo, Jacques Duranteau, Markus B. Skrifvars, Carolina Iaquaniello, Susanne Muehlschlegel, Victoria Metaxa, Claudio Sandroni, Giuseppe Citerio, Geert Meyfroidt

**Affiliations:** 1 Anesthesia and Intensive Care, San Martino Policlinico Hospital, IRCCS for Oncology and Neurosciences, Genoa, Italy; 2 Department of Surgical Sciences and Integrated Diagnostics, University of Genoa, Genoa, Italy; 3 Department of Acute Brain and Cardiovascular Injury, Mario Negri Institute for Pharmacological Research IRCCS, Milan, Italy; 4 Department of Critical Care, King’s College Hospital NHS Foundation Trust, London, SE5 9RS UK; 5 Departments of Neurology and Biomedical Informatics, Columbia University Vagelos College of Physicians and Surgeons, New York, NY USA; 6 Department of Intensive Care Medicine, Hôpital Bichat-Claude Bernard, Université Paris Cité, INSERM UMR 1137, IAME, APHP.Nord, Paris, France; 7 Neurological Intensive Care Unit, Department of Neurology, Medical University of Innsbruck, Innsbruck, Austria; 8 Department of Neurology, Johannes Kepler University, Linz, Austria; 9 Clinical Research Institute Neuroscience, Johannes Kepler University, Linz, Austria; 10 Wake Forest Baptist Health Center, Winston-Salem, NC USA; 11 PACE Section, Department of Medicine, University of Cambridge, Cambridge, UK; 12 Department of Intensive Care Adults, Erasmus MC-University Medical Centre, Room Ne-415, PO BOX 2040, 3000 CA Rotterdam, The Netherlands; 13 Department of Intensive Care Medicine, University Hospitals Leuven, Leuven, Belgium; 14 Laboratory of Intensive Care Medicine, Department of Cellular and Molecular Medicine, KU Leuven, Leuven, Belgium; 15 Department of Anaesthesia and Intensive Care, Centre Hospitalier Universitaire Grenoble, Universitaire Grenoble Alpes, Grenoble, France; 16 INSERM U1216, Grenoble Institut Neurosciences, Grenoble, France; 17 Department of Anaesthesiology and Critical Care Medicine, Bicêtre Hospital, Université Paris-Saclay, Assistance Publique des Hôpitaux de Paris, Équipe DYNAMIC, Inserm UMR 999, Le Kremlin-Bicêtre, France; 18 Department of Emergency Care and Services, University of Helsinki and Helsinki University Hospital, Helsinki, Finland; 19 Neuroanesthesia and Intensive Care, Department of Neurosurgery, Fondazione IRCCS Istituto Neurologico Carlo Besta, Milan, Italy; 20 Division of Neurosciences Critical Care, Departments of Neurology and Anesthesiology/Critical Care Medicine, Johns Hopkins University School of Medicine, Baltimore, MD USA; 21 Department of Intensive Care, Emergency Medicine and Anaesthesiology, Fondazione Policlinico Universitario A. Gemelli, IRCCS, Rome, Italy; 22 Institute of Anaesthesiology and Intensive Care Medicine, Università Cattolica del Sacro Cuore, Rome, Italy; 23 School of Medicine and Surgery, University of Milano-Bicocca, Monza, Italy

**Keywords:** Acute brain injury, Outcome, Prognostication, Neuroimaging, Neuromonitoring

## Abstract

Acute brain injuries, such as traumatic brain injury and ischemic and hemorragic stroke, are a leading cause of death and disability worldwide. While characterized by clearly distict primary events—vascular damage in strokes and biomechanical damage in traumatic brain injuries—they share common secondary injury mechanisms influencing long-term outcomes. Growing evidence suggests that a more personalized approach to optimize energy substrate delivery to the injured brain and prognosticate towards families could be beneficial. In this context, continuous invasive and/or non-invasive neuromonitoring, together with clinical evaluation and neuroimaging to support strategies that optimize cerebral blood flow and metabolic delivery, as well as approaches to neuroprognostication are gaining interest. Recently, the European Society of Intensive Care Medicine organized a 2-day course focused on a practical case-based clinical approach of acute brain-injured patients in different scenarios and on future perspectives to advance the management of this population. The aim of this manuscript is to update clinicians dealing with acute brain injured patients in the intensive care unit, describing current knowledge and clinical practice based on the insights presented during this course.

## Introduction

The management of acute brain injuries in the intensive care unit (ICU) remains a challenge. Despite considerable progress made over the past decades in terms of survival, many of these conditions continue to cause significant morbidity and mortality. In September 2023, the Neurointensive Care (NIC) section of the European Society of Intensive Care Medicine (ESICM) organized a comprehensive 2-day course to address the challenges in the managing patients suffering from acute brain injury (ABI). This manuscript aims to synthesize key concepts of the current standard clinical practice (including neuromonitoring techniques, pharmaceutical interventions, and neuroprognostication) discussed during this course, and relate these concepts to the underlying neurophysiology. Delving into the intricate relationship between glucose, lactate, and ketones, and exploring their derangements in the injured brain can help clinicians to optimize a nutrient supply to their patients. In addition, exploring brain-organ crosstalk in ABI unveils interconnected entities crucial during critical conditions. The manuscript further navigates the uncertainties of neuroprognostication, underscoring the challenges in presenting correct information to patients and families for informed decision-making. Finally, it explores potential future treatment strategies at the horizon to master the brain in critical conditions.

## Basic neurophysiology and neuropathophysiology

**Fig. 1:**
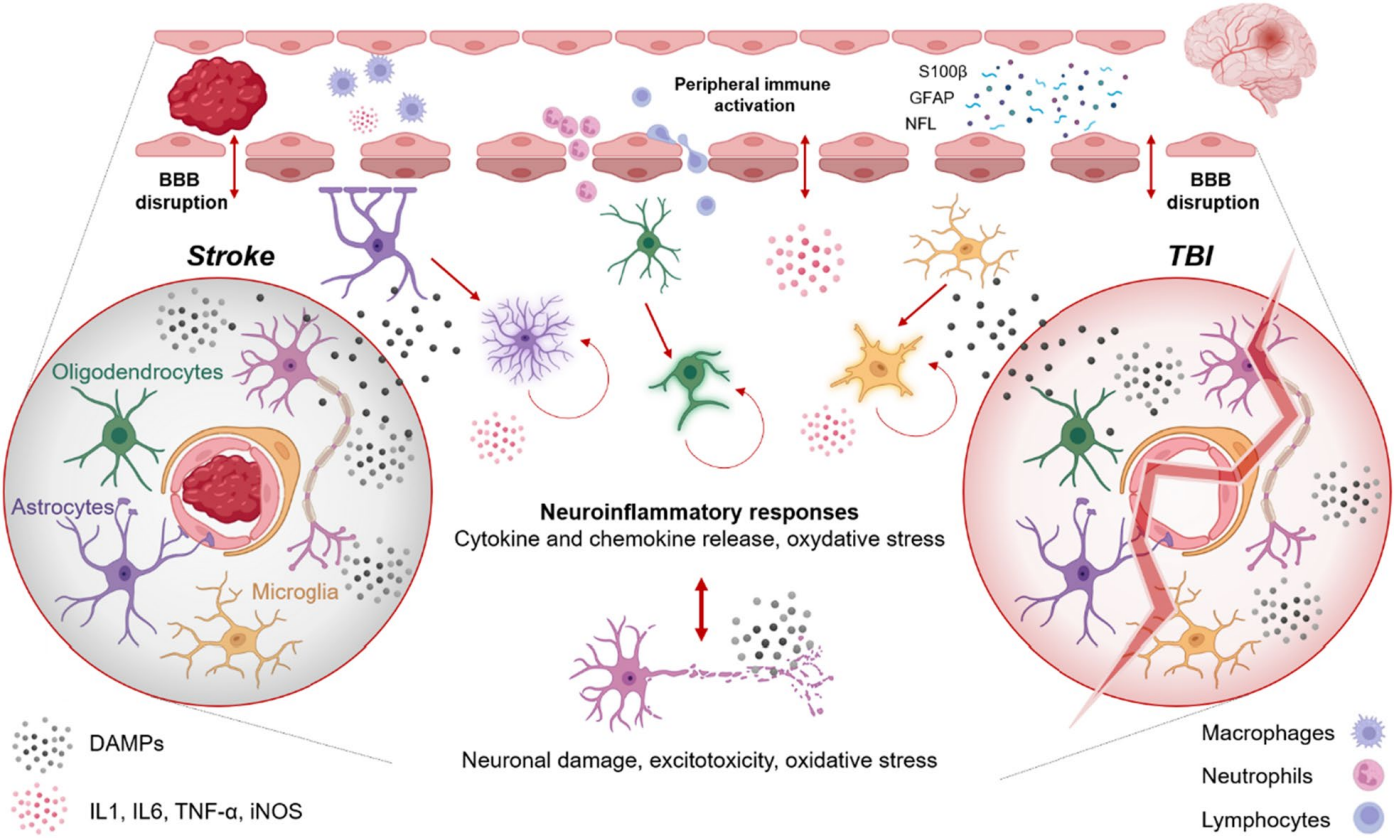
Neuroinflammatory response after ABI. Inflammation triggered by release of damage-associated molecular patterns (DAMPs) and reactive oxygen species (ROS) will cause activation of resident immune cells and release of proinflammatory citokines. Invasion of the CNS by circulating immune cells initially consists of innate immune cells, but is joined by adaptive leukocytes within days (primed T cells). While this response largely wanes, a proportion of patients display a persistent immune dysregulation, directly contributing to tissue damage. *BBB* blood brain barrier, *DAMPS* damaged associated molecular patterns, *IL* interleukin, *iNOS* inducible nitric oxide synthase, s100 calcium binding protein B (s100B); glial fibrillary acid protein (GFAP) neurofilament light (NFL); *TGF* transforming growth factor, *TNF* tumor necrosis factor. Figure created with BioRender.com

The human brain is the most intricate and irreplacable organ in the human body. It is physically protected by complex layers of physical barriers, encompassing the skin, the cranial vault, and the meninges. Comprising over 100 billion neurons, the brain operates as a highly intricate biological system, orchestrated by a sophisticated network of processes driven by action potentials and neurotransmitters. These intricate operations demand much energy, requiring a substantial and uninterrupted provision of glucose and oxygen to maintain functionality [1]. It is worth noting that glial cells play pivotal roles in these processes, in addition to their immune functions. In contrast to the brain's physical “protection”, its vulnerability due to negligible energy reserves is striking, making it highly dependent on a continuous supply of glucose and oxygen for function and survival [2]. Acute brain injuries, such as stroke or traumatic brain injury (TBI), are capable of inducing an imbalance between energetic supply and metabolic needs with profound consequences. The release of endogenous danger signals after ABI induces an innate ad adaptative immune activation, that is key to brain recovery; however, a dysregulated immune response can contribute to poor outcome (Fig. 1) [3]. These immune dynamic processes persist for months and even years and are governed by extracellular injury signals and intracellular molecular pathways. The ‘core and penumbra’ concept illustrates how the initial injury extends into neighboring regions, initiating a cascade of molecular events [4, 5]. Understanding these enduring molecular alterations reveal a significantly larger therapeutic window than previously thought, both in terms of time and spatial extent. Recognizing the long-lasting molecular alterations in the wake of ABI opens new avenues for therapeutic interventions that extend well beyond the immediate injury event. Recent research has highlighted the multifaceted roles of microglia within both normal brain development and the context of pathological conditions, shedding light on the potential for interventions that target these long-term molecular changes [2].

## Aging and gender effects in ABI

The association between age and ABI is complex, and influenced by physiological vulnerabilities and other risk factors. Advancing age increases susceptibility to several ABIs. Age-related cardiovascular risk factors may render the brain more susceptible to cerebrovascular diseases. In addition, older individuals often experience an increased risk of falls, which can lead to severe TBI. Pre-existing health conditions or medications can exacerbate the severity of brain injuries, as often seen in structural hemorrhagic lesions associated with the use of anticoagulants and antiplatelets agents. Pre-injury risk factors often observed in older individuals, such as frailty or dementia, have an obvious impact on the ability to withstand and recover from cerebral insults [6]. The aging process also affects the body's overall resilience, including compromised immune responses and diminished cellular repair mechanisms.

Sex differences should also be taken into account when managing patients with ABI. Besides anatomical, physiological and hormonal differences, comorbidities identified at ICU admission may differ between men and women [7]. Sex influences the risk of ABI with women exhibiting a higher incidence of subarachnoid hemorrhage (SAH) at all ages. In addition, in later life, women also have a higher incidence of TBI [8, 9]. However, their under-representation in clinical trials and cohort studies remains a concern. Sex and gender differences may also influence clinicians’ decision making and treatment. Understanding the intricate relationship between age, sex/gender and ABI is crucial for developing targeted interventions. It can be hypothesized that treatment strategies tailored to different age and sex groups could ultimately improve outcomes and quality of life for individuals across the lifespan, despite no differences in treatment currently being recommended.

## Clinical evaluation of neurocritical ill patients

ESICM guidelines recommend daily neurological examination as an integral part of the assessment of neurocritically ill patients. In the prehospital and acute phase, it is a limitation of the Glasgow Coma Scale (GCS) that brain stem reflexes including pupillary responses [10] are not evaluated. The Full Outline of UnResponsiveness (FOUR) score [11], which assesses motor and eye responses, brainstem reflexes and respiratory pattern, may overcome these limitations.

Despite the use of sedatives, a structured neurological exam can determine nature and severity of neurological dysfunction in the acute phase, helping to establish a plan for further diagnostics and treatment. In non-traumatic coma, the value of a full neurological examination to detect focal deficits as indicators for cerebral causes of acute coma has been studied [12]. In both populations, combining pupil size, light reaction, gaze, and pyramidal signs has shown better sensitivity and specificity than if used in isolation. Still, some focal brain pathologies may be overlooked if located in non-eloquent areas of the brain, making neuroimaging an important adjunct [10]. In the absence of focal signs in acute coma, complete work-up should include an in-depth history, laboratory tests and neuroimaging.

Pupillary reaction has been classically assessed using penlights. Recent data suggest this method does not accurately assess pupillary function [13]. Conversely, automated pupillometry is reliable and yields reproducible results. Pupillometry has become standard of care in many ICUs due to its accuracy in evaluation and predicting outcome [13]. Using pupillary reactivity in combination with individual motor response has proved to outweigh the full GCS in predicting outcome [14]. In the subacute phase, a structured neurological examination can determine prognosis and identify patients who can benefit from neurorehabilitation.

Throughout the ICU stay, confounders such as medications, delirium and metabolic or physical disturbances should be considered when interpretating neurological exam in populations, such as the elderly population, where drug metabolism is affected by organ dysfunction and altered pharmacokinetics and dynamics [15].

## Diagnostic and therapeutic conundrums in coma of unknown origin

Coma of unknown origin is a medical emergency than can be due to structural brain lesions, diffuse neuronal dysfunction, and, rarely, psychiatric causes [16, 17] (Table 1). Following stabilization and supportive care, more so in the absence of TBI or cardiac arrest, clinicians must employ a detective-like approach to identify the underlying issue and treat potentially reversible causes. A thorough neurological and general examination must be conducted and clues to reversible factors (intoxication, metabolic issues) can be best found with a detailed medical history from family, witnesses, or records [18]. Physical examination targets neurological signs, vital parameters, and evidence of trauma, integrated by laboratory tests (glucose, electrolytes, toxicology). Subsequent investigations should align with diagnostic hypotheses, and a table summarizing investigations is proposed below. Non-contrast CT scan diagnose major neurosurgical emergencies, while CTA rules out the rare but reversible basilar artery occlusion as a cause of coma. EEG rules out non-convulsive seizures and evaluates the cerebral electrical activity. Lumbar puncture may be needed in case of suspected CNS infections or neuroinflammatory diseases. Rarer metabolic causes (e.g., hyperammonemia, adrenal insufficiency, hypothyroidism) should be systematically explored in patients with coma of unclear origin. Multidisciplinary care involving neurologists, intensivists, radiologists, and infectious disease specialists ensures timely management and prognostication. The outcome remains uncertain in some cases, with potential complications hinging on underlying pathology and timely care. During ICU stay, repeated consciousness monitoring should be done using internationally recognized scales, such as the GCS or FOUR score.

**Table 1:** Primary investigations in case of coma of unknown origin

Test	Indication	Scope
Neurological examination, temperature, pulse, BP, EKG, RR, SpO_2_	All patients	
*Blood*		
Blood glucose	All patients	Rule out hypoglycemia
Electrolytes	All patients	Rule out severe hypo- or hypernatremia
Blood gas analysis	All patients	Rule out hypercapnia
Blood cultures	On clinical suspicion	Rule out systemic infection
Toxicology screening (from blood and urine)	On clinical suspicion	Rule out intoxications
Ammonemia and liver function	On clinical suspicion	Rule out hepatic encephalopathy
Cortisol	On clinical suspicion	Rule out adrenal insufficiency
Thyroid function	On clinical suspicion	Rule out hypothyroidism
*CSF*: routine cell count, protein, Glucose, Gram staining, India ink stain; cultures, including tuberculosis and fungal agents; cytology**.** HSV and VZV PCR; other agents depending on presentation	On clinical suspicion	Rule out CNS infections, neuroinflammatory diseases or cancer and leukemia dissemination
*Non-contrast CT*	All patients	Diagnosis of neurosurgical emergencies and massive stroke
*CTA*	On clinical suspicion	Rule out basilar artery occlusion
*MRI*	On clinical suspicion	Indicated in case of brainstem symptoms, unexplained coma, or suspected encephalitis
*EEG*	On clinical suspicion	Rule out nonconvulsive status epilepticus; may identify electrical patterns typical in some etiologies (e.g., triphasic waves in metabolic encephalopathy)

*CNS* central nervous system, *CT* computer tomography, *CTA* computed tomography angiography, *EEG* electroencephalogram, *GCS* Glasgow coma scale, *FOUR* Full Outline of UnResponsiveness coma scale, *TBI* traumatic brain injury

## Unlocking the potential: neuromonitoring for informed bedside clinical decision-making

**Fig. 2:**
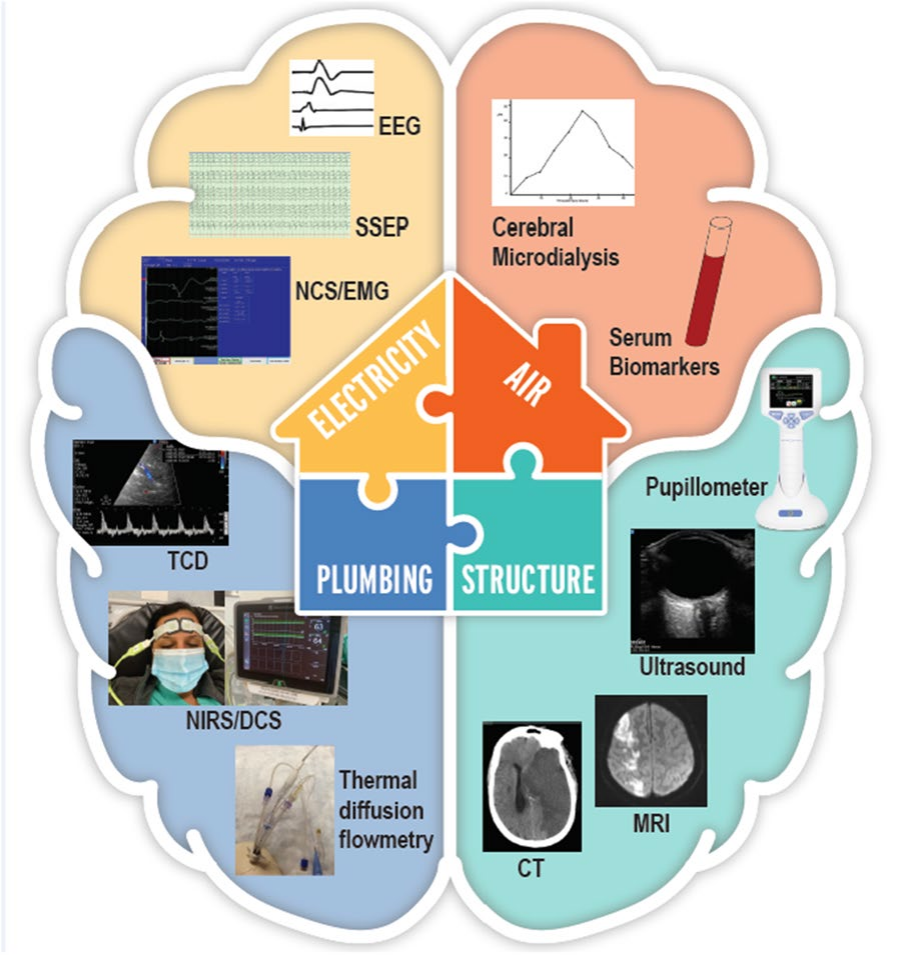
Conceptual framework of various neuromonitoring devices based on the model of “a house”. Structural assessments of brain parenchyma include pupillometry and neuro-radiology modalities, such as computed tomography (CT) and magnetic resonance (MR) imaging. Plumbing or cerebral hemodynamic assessments include brain tissue perfusion monitors, transcranial Doppler, and near-infrared spectroscopy. This may also include vascular Imaging such as CT angiography and CT perfusion (not shown) and intracranial pressure monitors. An assessment of the electrical compartment may include electroencephalography,somatosensory evoked potentials and nerve conduction velocity/electromyography. Assessment of neurochemistry make constitute the last compartment, including cerebral micro dialysis and serum and cerebro spinal fluid biomarkers. Copyright permission from Rajagopalan S, Sarwal A. Neuromonitoring in critically ill patients. Crit Care Med. 2023 Apr 1;51(4):525–542

The possibilities to monitor the brain are very extensive (Fig. 2). While clinical neurological examination remains the gold standard to detect, diagnose, and follow-up on neurological conditions, also in critical care, patients with severe neurological deficits at baseline or those requiring sedation pose a particular challenge when using this as a surveillance tool for detecting neurological deterioration [19]. Critical care neuromonitoring strategies should be applied to support strategies for the prevention of secondary brain injury in patients presenting with neurological disease and the prevention of primary brain injury in general ICU [20]. A range of neuromonitoring modalities are available for clinical bedside decision-making. The non-invasive strategies range from pupillometry to ultrasonography, electroencephalography, near-infrared spectroscopy, and non-invasive intracranial pressure monitors that use micromotions of the skull [19]. Invasive strategies cover a wide variety of signals, including intracranial pressure (ICP) monitoring (via external ventricular drainage, or intraparenchymal probes), brain tissue perfusion monitoring, brain tissue oxygen monitoring, jugular venous oxygen saturation monitoring, or cerebral microdialysis. In addition, most of these devices allow for serial or continuous calculations of the cerebral hemodynamic status through assessment of cerebral perfusion pressure and cerebral autoregulation, two important physiological targets in brain-targeted resuscitation.

Current guidelines and expert consensus in various forms of neurological disorders recommend a multimodal approach to evaluation, management, and prognostication in critical care [21, 22]. Several multimodality monitoring paradigms have been proposed combining the above invasive and noninvasive strategies to guide goal-directed therapies and individualize neuromonitoring modalities and targets [22, 23]. The interpretation of such multitude of signals is complex, but at the same time the large amounts of data created by multimodality neuromonitoring are very well-suited to big data research techniques. Indeed, artificial intelligence has the potential to optimize bedside data visualization, create early warning systems, and bedside decision support. Several of such systems are currently being developed, but whether they can contribute to improve clinical outcomes remains to be proven in clinical trials.

## The evolving role of neuro-imaging in neurocritical care

Neuroimaging is crucial, not only in the diagnosis of brain injuries and secondary insults in the ICU but also for prognostication and understanding the underlying pathophysiology.

Computed tomography (CT) enables rapid assessment of brain pathology requiring acute treatments, and in combination with clinical assessment it can have clear prognostic value. Its ease of use, speed and wide availability means it remains the imaging modality of choice in the hyperacute phase for many conditions. Addition of contrast can facilitate imaging of the cerebral vessels as well as perfusion scans to look for areas of ischemia and/or blood brain barrier leak.

**Fig. 3:**
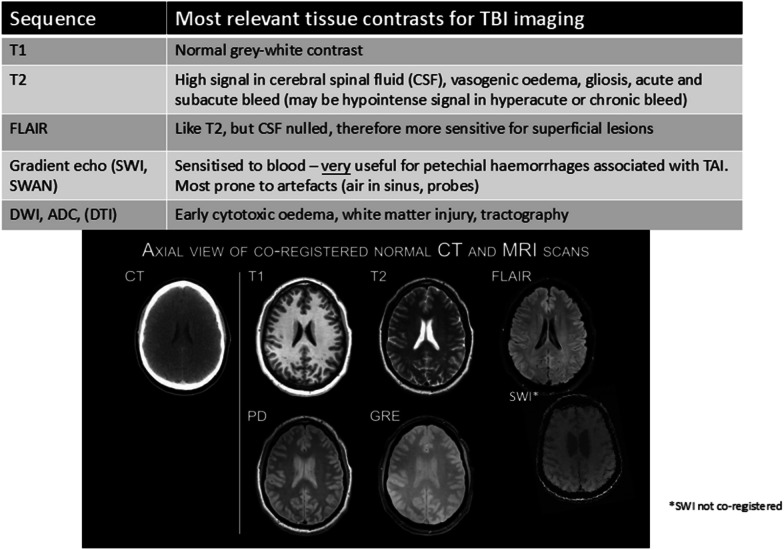
Clinical magnetic resonance imaging sequences: examples of magnetic resonance imaging sequences commonly used for traumatic brain injury

Magnetic resonance imaging (MRI) has higher resolution enabling detection of lesions, especially in the posterior fossa and deep white matter, with more advanced settings, including diffusion-weighted imaging and functional MRI facilitating greater insight into pathophysiology (Fig. 3) [24, 25]. The transfer of ventilated patients for MRI, however, is more challenging. Knowledge of how to use critical care equipment in MRI in safe, compatible ways is important. MRI provides more refined prognostic information for many conditions compared with CT including in TBI [26, 27] and ischemic–hypoxic brain injury secondary to cardiac arrest [28]. The exact location and type of injury is important and may be underappreciated, since for instance brainstem lesions in arousal centers have different prognostic implications to lesions elsewhere, and lesion characteristics (e.g., bleeding, infarction or oedema) are also important to consider [29]. Covert consciousness, or cognitive motor dissociation (CMD), may be detected using functional MRI in patients with prolonged disorders of consciousness [30].

After damage to the brain, proteomic biomarkers are released into the blood (Fig. 1), including astroglial biomarkers s100 calcium binding protein B (s100B) and glial fibrillary acid protein (GFAP), the neuronal biomarker neuron-specific enolase (NSE) and the axonal neurofilament light (NFL). Their early concentration scales with the extent of injury after TBI [31], and may help select patients who benefit most from MRI for detection of CT-occult lesion [32].

Neuroimaging after a brain injury is dynamic, with visible lesions progressing over time, which in some cases may no longer be visible in the subacute phase [33]. Pseudonormalisation is particularly important to consider with diffusion weighted imaging, and in particular pathologies, including hypoxic brain injury, where imaging performed in the subacute phase prior to atrophy may be normal or near normal despite significant injury [34]. In addition, the trajectory of ongoing white matter loss after TBI is associated with differing functional trajectories [35], and NFL concentrations in the early chronic phase are predictive of ongoing atrophy years after injury [36]. Such insights, while not currently used in clinical practice, show the potential for neuroimaging to understand ongoing pathophysiology, to potentially guide future management strategies and stratify patients for clinical trials.

## Glucose, lactate, ketones: when and how to feed the injured brain?

The brain accounts for 25% of the total body glucose consumption, with glucose being the main energy substrate for the brain in physiological circumstances [37]. Traditionally, elevated blood glucose evoked by stress has been considered adaptive, to ensure sufficient substrate for cells with insulin-independent glucose uptake, including neurons and astrocytes [38]. However, severe hyperglycemia associates with poor outcome in patients with ABI. Moreover, hyperglycemia can damage the brain, even in the absence of primary brain injury [39]. The ideal blood glucose target in critical illness has been debated, as also hypoglycemia should be prevented. The mortality benefit of tight glucose control in pioneer randomized controlled trials (RCTs) has been attributed to avoiding iatrogenic severe hyperglycemia evoked by early parenteral nutrition [40]; a feeding strategy that was abandoned after RCTs showed harm as compared with withholding parenteral nutrition in the first week in ICU [41, 42]. Recently, a large RCT revealed less severe hyperglycemia in patients not receiving early parenteral nutrition, and further lowering blood glucose to normal levels with a protocol that avoided hypoglycemia slightly improved morbidity without impacting mortality [40]. Yet, the intervention suggested possibly lower mortality in the subgroup of neuro-ICU patients, although the potential mechanisms remain to be investigated [40].

Potentially superior alternative energy substrates for the injured brain include lactate and ketones [43, 44]. Hypertonic lactate is an alternative substrate that may improve cerebral perfusion and increase cerebral glucose availability [44]. Ketones may additionally stimulate cellular repair processes, and part of the benefit of withholding early parenteral nutrition has been attributed to enhanced ketogenesis [45]. Although ketogenic diets have been successfully used in chronic epilepsy and refractory status epilepticus [43], large RCTs that investigated the efficacy and safety of ketones, ketogenic diets and hypertonic lactate in brain-injured patients are lacking.

## Interconnected entities: brain–organ crosstalk in ABI

Beside the brain, failure of other organs occurs frequently in the setting of ABI. In the first place, because the initial pathology leading to ABI may simultaneously cause injury to other organs. In trauma, for example, 55% of head-injured patients admitted to an ICU have concomitant major extracranial injuries (Abbreviated Injury Scale ≥ 3), most commonly involving chest (35%), spine (18%) and abdomen (17%) [46]*.* Hypoxemia, possibly due to pneumothorax, hemothorax, or pulmonary contusions following chest trauma, as well as arterial hypotension due to severe bleeding following spleen or liver injury, are well-known systemic secondary brain insults that will exacerbate the initial brain injury.

Second, neurological failure following brain injury may harm other organs. Impaired consciousness, resulting in loss of airway protective reflexes, can lead to aspiration pneumonia, and acute respiratory distress syndrome (ARDS). Regarding the cardiovascular system, ABI and the sudden increased intracranial pressure (ICP) can cause a sympathetic storm with a massive increase in catecholamine levels, resulting in myocardial contractility dysfunction, including Takotsubo cardiomyopathy [47]. This is an example of inter-organ crosstalk, which is a complex biological communication between remote organs mediated via cellular, molecular, metabolic and neurohormonal pathways. After ABI, primary damaged cells (neurons, astrocytes and other glial cells) release nuclear and cytoplasmic proteins that function as damage-associated molecular patterns (DAMPs). This triggers a strong innate and adaptive immune inflammatory response with activation of proinflammatory cascades in the injured brain and in remote organs. Activation of the hypothalamic–pituitary–adrenal axis system and the autonomic nervous system (sympathetic and parasympathetic) will release neurotransmitters and hormones (cortisol, catecholamines), inducing harm to remote organs when excessive or unbalanced [48]. Alterations to a remote organ may have, in turn, proper consequences to homeostasis. For example, ABI triggers dysbiosis of the gut microbiota characterized by a loss of commensal bacteria (*Firmicutes*, *Bacteroidetes*) and an increase in pathogenic bacteria (*Proteobacteria*). Indirect passage through the blood or lymphatic system of these gut bacteria may induce enrichment of the pulmonary microbiota and, in addition to mechanical ventilation, antibiotic use, diet changes and alterations of lung immunity, lead to hospital-acquired pneumonia and ARDS [48].

Treating one injured organ or system may lead to therapeutic conflicts, as illustrated by the example where increasing the blood pressure target to maintain cerebral blood flow could increase the afterload of the heart and impair cardiac function. High positive end-expiratory pressure (PEEP), permissive hypercapnia due to lung protective ventilation and recruitment maneuvers may increase ICP in a brain-injured patient with ARDS [49]. Long term consequences are also suspected, especially regarding the proinflammatory cascades triggered by ABI, as neuroinflammation plays a crucial role in neuroplasticity and brain recovery. Dysbiosis of the gut microbiota might be a possible cause of persistent and chronic disability in ABI, although the association with neurological outcome remains controversial. Future research may help to modulate these inter-organs pathophysiological processes, preventing progression to multiorgan failure, solving therapeutic conflicts and, hopefully, improving long-term recovery from brain damage.

## Alcohol: an unexpected neuroprotectant in ABI?

Chronic alcohol abuse and acute alcohol intoxication have several effects on the management of patients with TBI [50]. First, chronic alcohol consumption is a risk factor for TBI, and many TBI patients are intoxicated in the acute phase. The prevalence of alcohol intoxication among TBI patients treated in the intensive care unit ranges from 20 to 60% [51]. The typical severely intoxicated patient will have mainly ground-level falls with severe TBI and a lesser likelihood of extracranial injuries [52]. Severe alcohol intoxication makes the diagnosis of TBI difficult and may be associated with treatment delays and alcohol-related complications, including withdrawal symptoms, seizures and Wernicke’s encephalopathy [50]. Moreover, pre-injury chronic alcohol abuse is associated with coagulopathy, increased risk of intracranial haemorrhagic progression and poor clinical outcome. Alcohol use after TBI has also shown to worsen rehabilitation outcomes and prognosis and increase the risk of additional head injuries. Severe alcohol intoxication may result in a decreased level of consciousness, which may confound the assessment of TBI severity [53]. One study suggested, not unexpectedly, that among patients with minimal TBI findings on brain CT, severe intoxication may itself explain a decrease in the Glasgow Coma Scale score [45]. Thus, a wise clinical approach is to have a very low threshold for brain CT screening in intoxicated patients presenting with altered mental status.

Observational studies have shown that the presence of moderate levels of alcohol in the blood of TBI patients could be associated with decreased mortality [54]. On the other hand, other studies suggest that this may be related to unmeasured confounders [50]. However, some experimental data exist suggesting potentially protective effects of high levels of alcohol, such as a decrease in the inflammatory response and a decrease in reactive oxygen species [55]. Currently, the evidence does not support the clinical use of alcohol as a neuroprotectant for severe TBI, which for now should be considered an interesting but unproven hypothesis.

## There is no crystal ball: uncertainty in neuroprognostication

In patients with severe ABI, precise and early neuroprognostication holds significant value for all stakeholders, facilitating medical interventions and mitigating decisional conflict and decision-regret clinician burnout [56]. Nonetheless, it remains intrinsically limited due to inherent uncertainties.

In post-cardiac arrest hypoxic–ischemic encephalopathy (HIE), distinct predictors of adverse outcomes have been documented. Yet, these studies often entail a self-fulfilling prophecy bias, due to providers’ knowledge of the index test results, which may result in early withdrawal of life-sustaining therapies (WLST), overestimating predictor precision through censoring bias [57]. Contemporary guidelines advocate for a minimum 72-h observation period before prognostication to diminish premature WLST, exclusion of confounding factors and, in the case of HIE, require at least two concordant test results [58]. Considering the limited reliable diagnostic tests for other types of severe ABI and the seldom incorporated ICU patient trajectory in existing models, clinicians should avoid nihilistic prognostication and personal biases. Where confounding variables exist, or precise diagnostic tools lack, extended observation is advised [58]. To diminish bias in neuroprognostication research, strategies, such as blinding outcome assessors and treatment team, where feasible, postponing prognostication, and studying predictors in populations unaffected by WLST are essential.

The accuracy of neuroprognostication in severe ABI is low. For HIE, available strategies prioritise predicting poor outcome with high specificity at the expense of sensitivity, which rarely exceeds 50–60% [13].

Presently, there is significant variability in conveying prognostic information to surrogate decision-makers, introducing bias and resulting in misunderstandings [59]. Continued research is needed to improve prognostic accuracy and standardise clinician–family dialogue to minimise miscommunications and disparities.

## Hope at the horizon? Future neuroprotective strategies in ABI

Neuroprotection encompasses a broad spectrum of interventions aimed at improving the outcome of patients after an ABI event. Its fundamental goal is to preserve and restore the integrity, function, and connectivity of the brain cells not irremediably damaged by the initial injury [60]. Despite significant progresses in medical science, the development of effective neuroprotective drugs or strategies for TBI and haemorrhagic strokes has remained elusive. Nevertheless, there is optimism surrounding reperfusion strategies, which have shown promising outcomes in the context of ischemic strokes. These reperfusion strategies, involving the timely restoration of blood flow to the brain, have demonstrated potential in rescuing at-risk neural tissue, and minimizing the extent of damage caused by ischemia.

However, the challenges encountered in phase 3 clinical studies focusing on neuroprotection underscore the critical need for the formulation of new and innovative research paradigms. This calls for advancements in the design of experimental medicine studies for the identification of druggable targets and the evaluation of novel drugs or therapeutic strategies. These efforts should not only aim to mitigate the detrimental cascades of events initiated by the acute brain damage but should also be tailored to address specific disease phenotypes, based on disease-specific approaches [61]. This more "precise" and individualized approach could be developed using big data analytics in conjunction with evidence from fundamental studies in animals, translational research, and clinical trials, thus providing a comprehensive understanding of the underlying mechanisms of neuroprotection and regeneration.

The implementation of well-structured trials, with a targeted focus on enriched populations exhibiting precise phenotypic characteristics, appears to be a promising strategy. Proteomics biomarkers of brain injury including NFL, GFAP, S100B, NSE, tau, UCH-L1, and more recently variation of glucose over time have been identified as key clinical descriptors of disease trajectories in TBI patients and suggesting they might be important in future clinical practice [62]. Identifying specific biomarkers or genetic profiles that correlate with varying responses to neuroprotective interventions could facilitate the stratification of patient populations, enabling personalized and more effective treatment approaches [63–65].

Furthermore, exploring innovative strategies to promote neurorepair and neuroregeneration should involve a deeper investigation into the established efficacy of various therapeutic modalities, including stem cell therapies, neurotrophic factors, and gene therapies. In particular, the role of mesenchymal cell infusion in promoting recovery of functions warrants thorough exploration and evaluation, considering its potential to modulate the inflammatory response, enhance tissue repair, and promote neuronal survival and synaptic plasticity [66].

Overall, the multifaceted nature of neuroprotection demands a comprehensive and interdisciplinary approach, integrating the latest advancements in neuroscience, pharmacology, genetics, and technology to pave the way for more effective and tailored interventions in the field of ABI.

## Conclusions

The pathophysiology of acute brain injury is complex and requires a profound assessment of cerebrovascular function, and multimodal neuromonitoring tools and techniques. In the ICU, attention should be paid to a strict evaluation and treatment of intra and extracranial complications, which can worsen secondary brain damage and patients’outcome. Currently, many potential protective strategies, along with neuroimaging, biomarkers, and neuromonitoring tools, have been proposed. This offers hope on the horizon for both patients and clinicians.

## Data Availability

Not applicable.
